# Ruptured ileocolic artery pseudoaneurysm after laparoscopic appendectomy for acute appendicitis

**DOI:** 10.1186/s40792-022-01538-y

**Published:** 2022-09-29

**Authors:** Junpei Takashima, Keizo Taniguchi, Ayaka Koizumi, Fumi Shigehara, Kenji Yamazaki, Daisuke Fujimoto, Fumihiko Miura, Hirotoshi Kobayashi

**Affiliations:** Department of Surgery, Teikyo University School of Medicine, Mizonokuchi Hospital, 5-1-1 Futago, Takatsu-Ku, Kawasaki, Kanagawa 213-8507 Japan

**Keywords:** Appendectomy, Delayed rupture, Laparoscopy, Pseudoaneurysm

## Abstract

**Background:**

A pseudoaneurysm of the splanchnic vessels is considered to be rare, and in particular, very few cases of pseudoaneurysm in the ileocolic artery are reported. Here, we report a case of rupture of a pseudoaneurysm of the appendicular branch of the ileocolic artery after laparoscopic appendectomy.

**Case presentation:**

A 52-year-old man was diagnosed as having phlegmonous appendicitis, and an emergency laparoscopic appendectomy was performed. Bleeding from the inter-appendicular ligament during detachment of adhesions was stopped by white coagulation and Z-suture, and the inter-appendicular ligament was treated. The postoperative course was uneventful, and there were no adverse events or findings suggestive of abscess formation. On postoperative day 30, he presented with a ruptured pseudoaneurysm of the appendicular branch of the ileocolic artery. A definitive diagnosis was made by computed tomography, and emergency interventional radiology was performed with hemostasis achieved by coiling. The patient’s postprocedure course was favorable, and he was discharged with no adverse events, such as intestinal ischemia.

**Conclusions:**

We experienced a case of delayed pseudoaneurysm rupture after laparoscopic appendectomy. Care must be taken when handling the appendicular artery during the procedure, and the potential for pseudoaneurysm formation should be considered at postoperative follow-up.

## Background

A pseudoaneurysm is pathologically defined as a rupture of the vascular wall and accumulation of hematoma in perivascular connective tissues. A pseudoaneurysm of the splanchnic vessels is considered to be rare. In particular, very few cases of pseudoaneurysm in the ileocolic artery have been reported. Here, we report a case of rupture of a pseudoaneurysm of the appendicular branch of the ileocolic artery after laparoscopic appendectomy.

## Case presentation

**Fig. 1:**
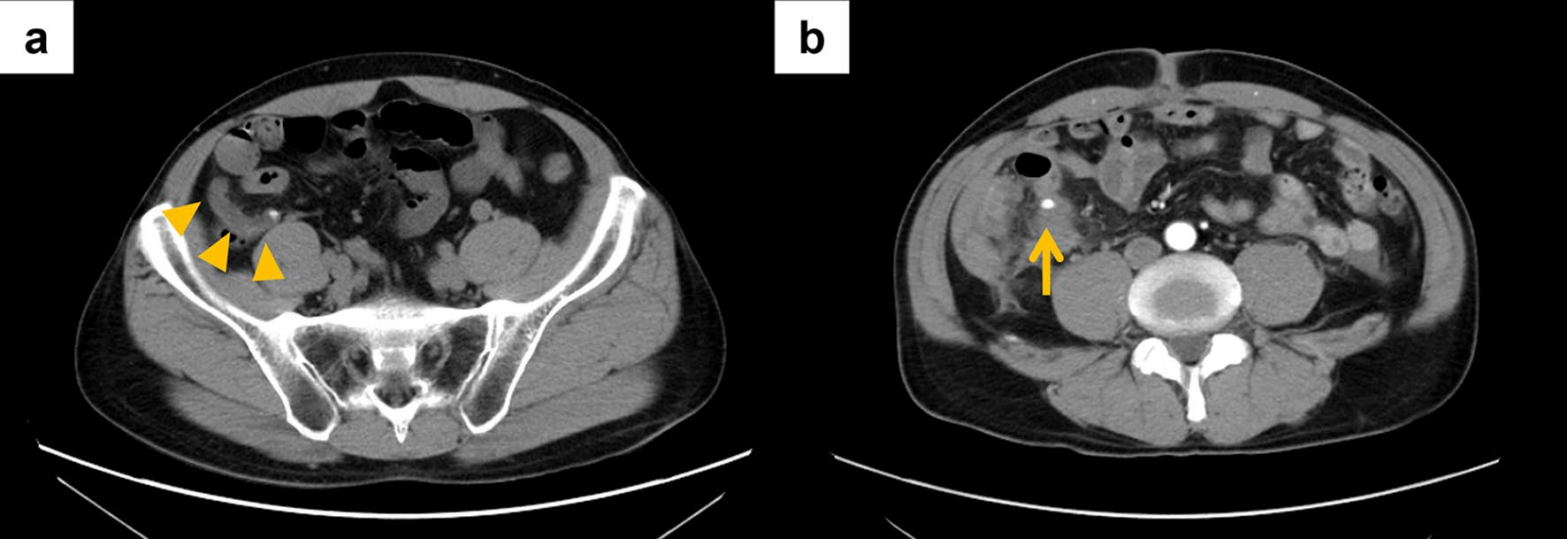
Abdominal contrast-enhanced CT findings. **a** At the first admission, a swollen appendix with a stercolith was observed at the root (arrowheads). **b** At the re-admission, a hematoma and a nodule suspected of being a pseudoaneurysm (arrow) were found in the ileocecal region

A 52-year-old man visited our hospital with right lower quadrant pain as his chief complaint. The patient had no past relevant history. He had experienced epigastric pain as a subjective symptom from the night before. The pain then moved to the right lower quadrant and gradually exacerbated. At the consultation, his vital signs were stable, and a low-grade fever was found. A physical exam found severe tenderness in the right lower quadrant. Blood tests showed an increase in white blood cells (WBC: 14.7 × 10^3^/μL) and a slight increase in C-reactive protein (CRP: 1.07 mg/dL), with other values within normal limits. Abdominal contrast-enhanced computed tomography (CT) revealed swelling of the appendix and a stercolith at the root of the appendix (Fig. 1a). He was diagnosed as having phlegmonous appendicitis, and thus, emergency laparoscopic surgery was performed.

**Fig. 2:**
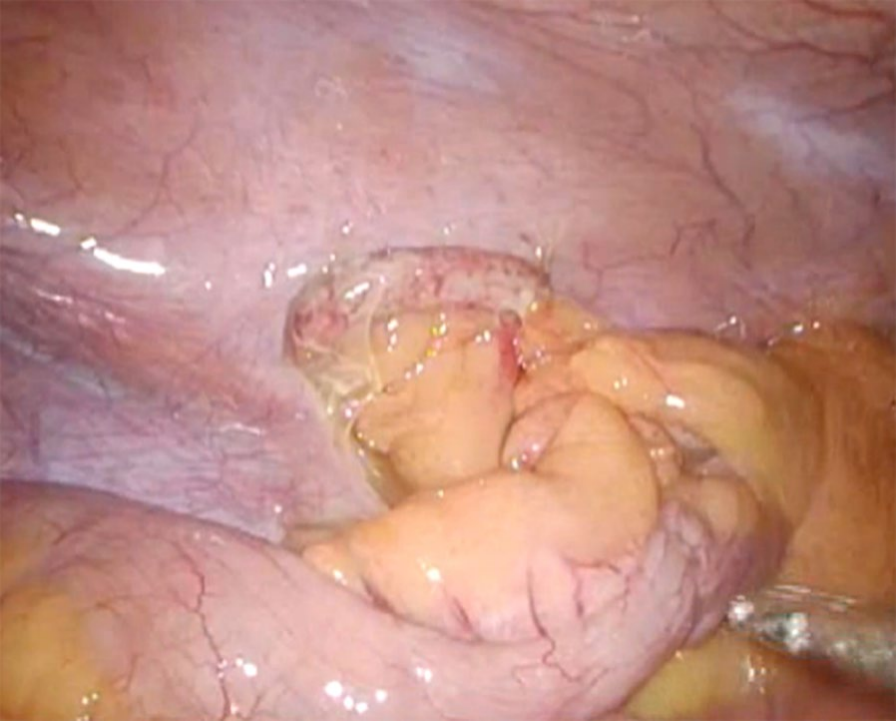
Intraoperative findings. At surgery, the appendix was swollen, and the thickened mesoappendix was adherent to the pelvic wall

The intraoperative findings showed a swollen appendix and slightly contaminated ascites fluid in the in the periphery of the appendix (Fig. 2). No abscess formation was found, and the findings were consistent with phlegmonous appendicitis. Adhesiotomy was performed to release the mesoappendix that was adherent to the pelvic wall. During this process, the mesoappendix began to hemorrhage. Therefore, white coagulation with a monopolar soft coagulation system (VIO, ERBE, Germany) and Z-suture technique were performed to obtain hemostasis. The division of the mesoappendix was performed proximal to the bleeding point with ultrasonic dissecting shears. Thereafter, a standard appendectomy was conducted to bury the appendicular root, and the operation was completed.

Postoperatively, the patient’s abdominal pain improved, and he could start taking meals from postoperative day (POD) 3 after admission. The postoperative course was free of adverse events, and he was discharged from hospital on POD 5. On POD 20, he visited our hospital outpatient clinic. Blood testing was negative for inflammatory reaction, the abdominal findings were normal, and the outpatient examination was completed. The pathological diagnosis was phlegmonous appendicitis.

On POD 30, he experienced sudden right lower quadrant pain in the afternoon and revisited the outpatient clinic. He had no fever, but his vital signs indicated shock (blood pressure: 91/65 mmHg; heart rate: 122/min). A physical exam found him in a cold sweat with localized severe tenderness in the right lower quadrant. Blood tests showed an increase in WBC (9.4 × 10^3^/μL) and a slight increase in CRP (0.91 mg/dL). However, no anemia was observed at this point. Abdominal contrast-enhanced CT revealed fluid accumulation in a high-density area from the ileocecum to the hepatic surface. A pseudoaneurysm was found near the ileocecum with extravasation (Fig. 1b). He was diagnosed as having post-appendicectomy ruptured ileocolic artery pseudoaneurysm, and emergency interventional radiology (IVR) was performed.

**Fig. 3:**
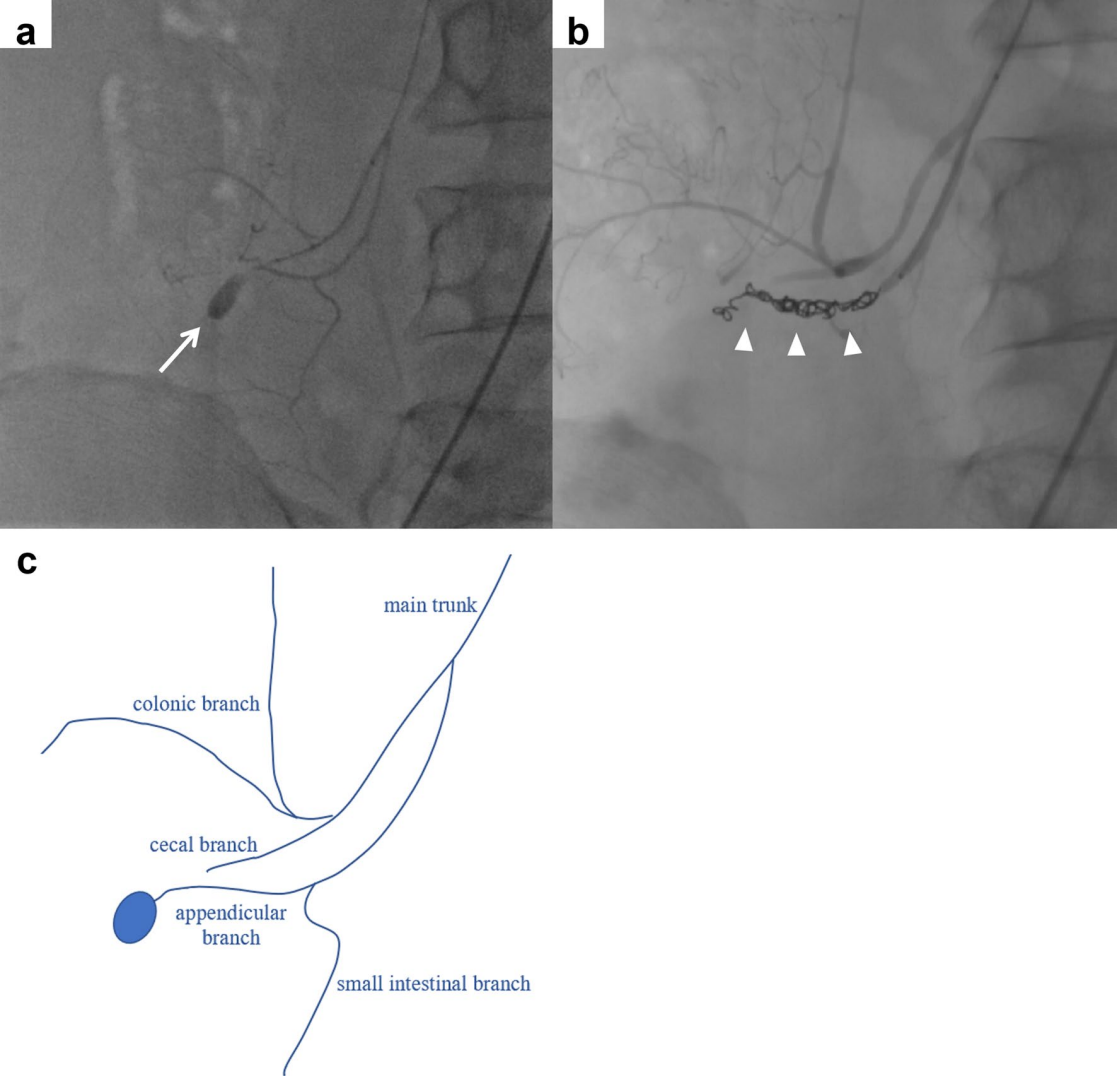
Interventional radiology findings. **a** Pseudoaneurysm was visualized in the appendicular branch of the ileocolic artery (arrow). **b** Coiling was performed from the pseudoaneurysm to the origin of the appendicular branch of the ileocolic artery, after which the pseudoaneurysm disappeared (arrowhead). **c** Illustration of the anatomy shown by angiography

When the iliac artery was catheterized from the superior mesenteric artery (SMA) and angiography was performed, a pseudoaneurysm was visualized in the periphery of the appendicular branch (Fig. 3a, c). A microcatheter was guided into the pseudoaneurysm, and a micro coil was inserted. Coiling was placed from the pseudoaneurysm to the root of the appendicular branch of the ileocolic artery to achieve hemostasis. A postoperative contrast-enhanced CT scan confirmed elimination of the pseudoaneurysm (Fig. 3b).

The patient’s post-IVR vital signs remained stable. On day 1 of admission, a decrease in his Hb to 7.5 g/dL was found, and 4 units of red blood cells were administered to improve the anemia. On POD 3, the patient began meals, and even after the patient started eating again, no findings suggestive of mesenteric ischemia were noted. The patient’s outcome was good, and he was discharged on POD 6. About 3 years have passed since discharge, and his course has been complication free.

## Discussion

A pseudoaneurysm may threaten life due to hemorrhage, and thus, an accurate and quick diagnosis is needed. Pseudoaneurysms of the splanchnic vessels are rare. Few cases have been reported collectively, and in a systematic review [1], only 15 cases of renal pseudoaneurysms reported by Ghoneim et al. [2] and 6 cases of hepatic pseudoaneurysms reported by Künzle et al. [3] were found. Pancreatitis is common in many cases, and more than 60% of pseudoaneurysms are said to be secondary to pancreatitis [4]. Other causes include abdominal trauma, iatrogenic trauma, vasculitis, and atheromatous, but their frequency is low [5]. Reports of pseudoaneurysm are very rare after an appendectomy. To the best of our knowledge, only two cases have been reported so far by Zia et al. [6]. The most frequently reported lesions were in the splenic vein, gastroduodenal artery, and pancreaticoduodenal artery [7], with only a few reports of pseudoaneurysm in the SMA. In the reports of postoperative cases of appendicitis, 1 case of right colic artery and 2 cases of ileocolic artery (including our own case) were included.

Pseudoaneurysm occurs as follows. Rupture of the arterial wall causes blood flow between the arterial lumen and perivascular connective tissues. A blood-filled cavity forms outside of the vascular wall, and leakage of blood is blocked by thrombus formation [8]. In pancreatitis, it is reported to result from the lack of arterial wall structures from the erosion of peripancreatic vessels caused by pancreatic enzymes and arterial wall erosion [9]. In cases of appendicitis, pseudoaneurysm may occur by the same principle if there is abscess formation. In our case, however, the postoperative course was good and did not raise any particular suspicion of abscess formation. Therefore, we suspected that the cause was due to handling of the appendicular artery during surgery and that the adventitia had ruptured due to insufficient vascular care. In addition, intraoperative findings showed that the mesoappendiceal ligament was severely thickened and was accompanied by strong inflammation that caused adhesion to the surrounding tissue. We surmised that the inflammation remaining in the mesoappendix affected the ruptured adventitia and led to the delayed onset of the pseudoaneurysm. Usually, when there is hemorrhage from elective laparoscopic surgeries for malignant tumors, such as gastric cancer, sufficient hemostasis is achieved with white coagulation, and no postoperative onset of pseudoaneurysm is found. There are two reasons for this. First, elective surgeries are usually not accompanied by inflammation. Even if the adventitia is ruptured, it is thought that inflammation will not spread there. Second, strong innervation surrounding the blood vessels treated for gastric and other cancers help to protect the vessels, whereas peripheral blood vessels such as the appendicular artery are less well-innervated, which might enhance aneurysm formation. Currently, at our facility, we have changed our procedure during laparoscopic appendectomy to conduct ligation of the appendicular artery as in laparotomy. Since this change, we have had no incidences of pseudoaneurysm.

While about 80% of true aneurysms in the visceral artery are asymptomatic, pseudoaneurysm is frequently symptomatic. The most serious complication of pseudoaneurysm is hemorrhage. The mortality rate of patients with hemorrhage is high at between 12.5% and 37% [10], and symptoms are inevitable in these cases. Common symptoms include abdominal pain, hematemesis/melena, and abdominal distension. Melena is particularly common, and one report indicated that gastrointestinal bleeding occurs in 89% of cases [11]. Including our case, the reported symptoms of post-appendicectomy pseudoaneurysm were melena in 1 case and abdominal pain in 2 cases. All three of these cases presented with strong subjective symptoms.

Angiography is reported to be the best-suited means of diagnosis, and real-time assessments with 100% sensitivity are possible. This is followed by CT (67%) and ultrasound (50%) [12]. Because pseudoaneurysms are generally at high risk for rupture, treatment is mandatory. In the past, surgical treatment was conducted for most cases [13], but in recent years, interventional angiographic treatment has become the accepted method. As it is minimally invasive and has a high success rate and low mortality rate, it is widely used to treat pseudoaneurysm [14]. In both our case and the cases reported by Zia et al. [6], pseudoaneurysm was suspected on CT imaging, and a definitive diagnosis and treatment were achieved by angiography. It is common to evaluate by CT at the time a pseudoaneurysm is suspected and then to perform angiography as a diagnostic treatment. There were some reports of post-embolization mesenteric ischemia occurring in cases of pseudoaneurysm [15], but in all three reported cases including our case, no symptoms of ischemia were found. For post-appendicectomy pseudoaneurysm, the culprit lesion is in a peripheral artery of the SMA branches, and selectively coiling would prevent symptoms of ischemia to a degree.

The final consultation for our patient was the outpatient examination on POD 20 after the first admission, but rupture of the pseudoaneurysm rupture was found on POD 30. For the other two reported cases, pseudoaneurysm rupture occurred 3 weeks after the operation and 36 h after the operation, respectively. When considering the risks of delayed pseudoaneurysm rupture, outpatient follow-up may need to be longer than usual if the appendix was highly inflamed and intraoperative treatment of the appendicular artery was difficult.

## Conclusions

We experienced a case of delayed rupture of a pseudoaneurysm of the appendicular branch of the ileocolic artery after laparoscopic appendectomy. Care must be taken when handling the appendicular artery during the procedure, and the potential for pseudoaneurysm formation should be considered at postoperative follow-up.

## Data Availability

Data sharing is not applicable to this article as no data sets were generated or analyzed during the current study.

## Abbreviations

CT: Computed tomography
CRP: C-reactive protein
IVR: Interventional radiology
SMA: Superior mesenteric artery
WBC: White blood cells
